# Transcutaneous auricular vagus nerve stimulation targets the brain-gut axis to treat functional dyspepsia: a scoping review of clinical evidence and mechanistic research

**DOI:** 10.3389/fnins.2026.1876591

**Published:** 2026-09-16

**Authors:** Xue Xiao, Xin Qin, Jing Tian, Xiaowen Chen, Xiang Luan, Yuren Sun, Jifei Sun, Yutang Ren

**Affiliations:** 1 Department of Psychiatry and Psychology, Beijing Tsinghua Changgung Hospital, School of Clinical Medicine, Tsinghua Medicine, Tsinghua University, Beijing, China; 2 Shunyi Hospital, Beijing Hospital of Traditional Chinese Medicine, Beijing, China; 3 Department of Gastroenterology, Beijing Tsinghua Changgung Hospital, School of Clinical Medicine, Tsinghua Medicine, Tsinghua University, Beijing, China

**Keywords:** brain–gut axis, functional dyspepsia, neuromodulation, scoping review, transcutaneous auricular vagus nerve stimulation

## Abstract

**Background:**

Functional dyspepsia (FD) is a common gut–brain interaction disorder with suboptimal response to pharmacotherapy. Transcutaneous auricular vagus nerve stimulation (taVNS), a non-invasive neuromodulation targeting the vagus nerve, has emerged as a promising brain–gut axis therapy. This scoping review (PRISMA-ScR, JBI methodology) synthesizes clinical and preclinical mechanistic evidence on taVNS for FD.

**Methods:**

PubMed, Embase, Web of Science, and Cochrane Library were searched up to December 20, 2025, following PRISMA-ScR. Using PICOS (adults with FD; taVNS; sham/non-vagal/active controls; symptoms, gastric function, psychological outcomes, biomarkers; RCTs/clinical trials and controlled animal studies), two reviewers independently screened, extracted data, and assessed bias (RoB2 for RCTs, SYRCLE for animal studies). A narrative synthesis was performed due to heterogeneity.

**Results:**

Four RCTs (*n* = 480) and eight preclinical rodent studies met inclusion. All RCTs showed significant improvements in dyspeptic symptoms, gastric accommodation, slow-wave normalization, and comorbid anxiety, depression, sleep disturbance versus sham/non-vagal control, sustained for 4–12 weeks. Stimulation parameters varied (10–30 Hz; 0.5–10 mA; 2–4 weeks); risk of bias low-moderate. Preclinical rodent evidence supported five interconnected candidate mechanisms: (i) autonomic balance and cholinergic modulation; (ii) α7nAChR/NF-κB anti-inflammatory pathway with duodenal barrier restoration; (iii) NGF/TrkA/PLC-γ/TRPV1-mediated visceral hypersensitivity suppression; (iv) HPA axis inhibition; (v) rebalancing of brain-gut peptides (motilin, CCK, GLP-1).

**Conclusion:**

This review characterizes taVNS as a safe, well-tolerated, mechanistically plausible non-invasive neuromodulation for FD acting on multiple brain–gut axis nodes. However, the small number of trials, parameter heterogeneity, and limited follow-up constrain definitive conclusions. Adequately powered, multicenter RCTs with standardized protocols and long-term follow-up are needed to confirm efficacy and define optimal regimens.

## 1 Introduction

Functional dyspepsia (FD) is a common disorder of gut–brain interaction, characterized clinically by upper abdominal discomfort or pain, bloating, early satiety, belching, and acid reflux (Zhong et al., 2024). It is the most prevalent of the functional gastrointestinal disorders, with a global prevalence of approximately 10%–30% (Sayuk and Gyawali, 2020). Only about 40% of patients with FD actively seek medical care when symptoms worsen or recur frequently (Ford et al., 2007). Although FD is not life-threatening, long-standing illness substantially affects patients’ physical and mental health and social functioning. FD is associated with increased risks of depression, anxiety disorders, and sleep disturbance (Rosca et al., 2025), and a subset of patients develop complications such as gastrointestinal bleeding and iron deficiency anemia (Gabbard and Vijayvargiya, 2024). Collectively, these factors markedly reduce quality of life and impose a substantial economic and societal burden.

Current treatments for FD face multiple limitations. Prokinetic agents, acid-suppressing drugs, and visceral sensitivity modulators are often ineffective in a substantial proportion of patients and show marked interindividual variability (Koochakpoor et al., 2024). Long-term medication use may also cause adverse effects–such as nutrient malabsorption and gut microbiota dysbiosis associated with acid suppressants–compromising treatment tolerability and safety (Liang et al., 2025). Furthermore, high rates of symptom recurrence after discontinuation suggest that current approaches focus primarily on symptom management rather than on addressing the underlying disease mechanisms (Huang et al., 2025). These limitations underscore an urgent clinical need for safer, longer-lasting, and more personalized treatment strategies.

The brain–gut axis is a bidirectional regulatory network that integrates the central nervous system, the enteric nervous system, the autonomic nervous system, and the endocrine–immune system (Cryan et al., 2019; Margolis et al., 2021). In FD, all components of this axis are dysregulated to varying degrees, including altered gut microbiota composition (Pan et al., 2023; Ju et al., 2023), heightened sensitivity of the enteric nervous system (Keohane and Quigley, 2006), abnormal central processing of afferent signals manifesting as excessive activation in the anterior cingulate cortex and insula (Liu et al., 2018), and low-grade mucosal inflammation with elevated pro-inflammatory cytokines (Zhang et al., 2025; Vanuytsel et al., 2023). These interconnected dysfunctions form the pathophysiological basis for persistent symptoms in FD and provide a rationale for therapies that act on the brain–gut axis as a whole, rather than on isolated downstream targets.

Transcutaneous auricular vagus nerve stimulation (taVNS) is a non-invasive neuromodulation technique that exploits the unique anatomical distribution of the auricular branch of the vagus nerve (Arnold’s nerve), which innervates the skin of the external auditory canal and concha–the only cutaneous representation of the vagus nerve (Kim et al., 2022; Liu C. et al., 2025). Electrical signals applied at this site travel via auricular vagal afferents and the jugular ganglion to the nucleus of the solitary tract (NTS) in the medulla, and from there are disseminated to monoaminergic brainstem nuclei (locus coeruleus, raphe nuclei) and forebrain regions including the hypothalamus, amygdala, and prefrontal cortex (Owens et al., 2024; Huang et al., 2023; Frangos et al., 2015; Breit et al., 2018). This multilevel pathway makes taVNS mechanistically well suited to modulating the dysregulated brain–gut axis in FD.

Preliminary clinical studies suggest that taVNS can improve gastrointestinal motility, alleviate visceral hypersensitivity, suppress mucosal inflammation, and modulate vagal tone, with concurrent benefits on comorbid anxiety, depression, and sleep disturbance (Li J. et al., 2025; Shi et al., 2024; Zhou et al., 2025). These early signals should nevertheless be interpreted conservatively. The randomized evidence base in FD is small–four trials in total, three of which enrolled fewer than 100 participants–and every trial published to date has been conducted in a single country (China), so that the generalizability of the reported effect sizes to other health-care settings, dietary patterns, and patient populations is untested. Stimulation parameters, sham design, and outcome instruments also differ markedly between trials, and no study has followed patients beyond 12 weeks; independent replication across research groups and devices is therefore still lacking. Accordingly, taVNS for FD is best regarded at present as a biologically plausible but methodologically immature intervention, and the purpose of the present review is to map that evidence base transparently rather than to advocate for the technique. However, the clinical and mechanistic evidence has not been systematically appraised. Previous reviews on this topic have been narrative in nature and have not applied transparent eligibility criteria, structured data extraction, or formal risk-of-bias assessment. As a result, the magnitude and consistency of therapeutic effects, the optimal stimulation parameters, and the relative contributions of individual mechanistic pathways remain unclear.

Comparison with other disorders of gut–brain interaction (DGBI) clarifies why FD warrants dedicated synthesis rather than extrapolation from adjacent conditions. taVNS has been applied across neurological, psychiatric, and gastrointestinal indications (Wang et al., 2021), but the maturity of the evidence differs substantially by disease. In irritable bowel syndrome (IBS), auricular neurostimulation has been evaluated by geographically independent groups: percutaneous electrical nerve field stimulation of the external ear reduced abdominal pain in adolescents with functional abdominal pain disorders and with IBS in double-blind, sham-controlled trials conducted in the United States (Kovacic et al., 2017; Krasaelap et al., 2020), whereas taVNS improved bowel-movement frequency, abdominal pain, rectal sensation, and psychological comorbidity in randomized trials of constipation-predominant IBS conducted in China (Shi et al., 2021; Liu C. et al., 2025). In gastroparesis, by contrast, the available clinical data derive from non-invasive cervical rather than auricular stimulation and consist of a proof-of-concept study and an open-label pilot without sham control, in which symptomatic and gastric-emptying benefit was confined to a subset of patients (Paulon et al., 2017; Gottfried-Blackmore et al., 2020). FD thus occupies a distinctive intermediate position: unlike gastroparesis, it has been examined with sham-controlled auricular protocols and with objective gastric physiological endpoints such as accommodation, slow-wave activity, and vagal efferent indices; unlike IBS, its clinical evidence has not yet been reproduced outside a single country, and its mechanistic account rests almost entirely on rodent models rather than on human physiological studies. Mapping the FD-specific literature–its parameters, comparators, endpoints, and mechanistic claims–is therefore a prerequisite both for designing the confirmatory, geographically diverse trials that the field now requires and for judging which observations in IBS may legitimately be extrapolated to the upper gut.

Following the PRISMA extension for Scoping Reviews (PRISMA-ScR; Tricco et al., 2018) and the JBI methodology for scoping reviews (Peters et al., 2020), this scoping review aims to (i) map the extent, range, and nature of the available clinical and preclinical evidence on taVNS for FD; (ii) summarize the reported clinical effects and the candidate mechanistic pathways through which taVNS modulates the brain–gut axis; and (iii) identify methodological gaps and priorities for future research, with particular attention to the parameters, comparators, outcome instruments, and translational endpoints that warrant standardization in subsequent definitive trials.

## 2 Methods

This scoping review was conducted and reported in accordance with the Preferred Reporting Items for Systematic Reviews and Meta-Analyses (PRISMA) 2020 statement (Page et al., 2021) and its extension for Scoping Reviews (PRISMA-ScR; Tricco et al., 2018), and was guided by the Joanna Briggs Institute (JBI) methodology for scoping reviews (Peters et al., 2020) building on the framework of Arksey and O’Malley (2005) and the methodological refinements of Levac et al. (2010). The completed PRISMA-ScR checklist is provided in Supplementary Material 1.

This work is submitted under the Systematic Review article type as a Scoping Review, following the Frontiers article-type definition which explicitly includes scoping reviews (alongside mapping reviews, meta-syntheses, meta-analyses, and systematic reviews) within this category. Reporting conforms to the relevant guidelines for scoping reviews: PRISMA-ScR for the overall reporting framework (Tricco et al., 2018), the JBI methodology for scoping reviews for protocol design and synthesis (Peters et al., 2020), and the methodological framework of Arksey and O’Malley (2005) as refined by Levac et al. (2010). Although a formal risk-of-bias appraisal is not required for scoping reviews under PRISMA-ScR, we additionally applied the Cochrane Risk of Bias 2 (RoB 2) tool to the included randomized clinical trials (Sterne et al., 2019) and the SYRCLE risk-of-bias tool to the included controlled animal experiments (Hooijmans et al., 2014) to enhance the transparency and rigor of the evidence map. As a Scoping Review, this article reports only previously published, peer-reviewed evidence and contains no unpublished data, submitted manuscripts, or personal communications.

Protocol registration. This review was not prospectively registered; consistent with current guidance, PROSPERO does not accept scoping-review protocols, and we did not register the protocol on the Open Science Framework (OSF), which is a limitation acknowledged below. To enhance transparency and reproducibility, the completed PRISMA-ScR checklist (Supplementary Material 1), the full database search strategies (Supplementary Material 2), and the detailed risk-of-bias assessments for every included study (Supplementary Material 3) are provided so that readers and reviewers can independently evaluate our methods.

### 2.1 Review question and PICOS framework

The review was structured around two complementary research questions addressing the clinical and mechanistic evidence on taVNS for FD. The PICOS framework for the clinical evidence is defined in Table 1. Although the Population–Concept–Context (PCC) framework is the formal mnemonic recommended by the JBI methodology for scoping reviews (Peters et al., 2020), we deliberately adopted the more granular PICOS structure to disambiguate intervention parameters, comparators, and outcomes of taVNS for FD; the PCC elements are fully captured within this PICOS specification (Population = adults with FD; Concept = taVNS or auricular vagus nerve stimulation; Context = clinical-effect synthesis and preclinical mechanistic synthesis).

**TABLE 1:** PICOS framework for the scoping review.

Domain	Definition
Population (P)	Adults (≥18 years) diagnosed with functional dyspepsia according to Rome III or Rome IV criteria, including postprandial distress syndrome (PDS), epigastric pain syndrome (EPS), and non-ulcer dyspepsia, with or without comorbid psychological symptoms or sleep disturbance.
Intervention (I)	Transcutaneous auricular vagus nerve stimulation (taVNS), transcutaneous vagus nerve stimulation (tVNS), or auricular vagus nerve stimulation delivered to the auricular branch of the vagus nerve (cymba conchae, cavum conchae, or tragus), with any stimulation parameters and any treatment duration.
Comparator (C)	Sham stimulation at non-vagal auricular sites (e.g., earlobe, auricular scapha), transcutaneous non-auricular vagus nerve stimulation (tnVNS), active pharmacological controls, no intervention, or waitlist control.
Outcomes (O)	Primary: composite dyspeptic symptom scores and clinical response/effective rate. Secondary: gastric accommodation, gastric emptying, proportion of normal gastric slow waves, vagal activity indices (HRV high-frequency power, LF/HF ratio), anxiety and depression scores, sleep quality, FD-related quality of life, and adverse events.
Study design (S)	For the clinical synthesis: randomized controlled trials (RCTs), non-randomized controlled trials, single-arm interventional studies, and case reports. For the mechanistic synthesis (secondary question): controlled preclinical experiments in rodent FD models.

### 2.2 Eligibility criteria

Inclusion criteria: (1) original peer-reviewed research articles; (2) studies enrolling participants who met recognized diagnostic criteria for FD or, for the mechanistic synthesis, rodents in validated FD models; (3) interventions involving taVNS, tVNS, or auricular vagus nerve stimulation; (4) any of the study designs specified in the PICOS framework; and (5) full text available in English.

Exclusion criteria: (1) non-original research formats, including narrative reviews, meta-analyses, commentaries, editorials, letters, and conference abstracts; (2) interventions that did not involve electrical stimulation of the vagus nerve; (3) studies that did not report relevant clinical or mechanistic outcomes; (4) studies for which essential data could not be obtained despite attempts to contact the corresponding author; and (5) studies not primarily focused on FD.

### 2.3 Information sources and search strategy

A systematic literature search was conducted in PubMed, Embase, Web of Science, and the Cochrane Library from database inception to 20 December 2025. The search strategy combined Medical Subject Headings (MeSH) with free-text keywords and was developed in consultation with all reviewers. Principal search terms and their combinations included “transcutaneous auricular vagus nerve stimulation,” “transcutaneous vagus nerve stimulation,” “auricular vagus nerve stimulation,” “taVNS,” “tVNS,” “dyspepsia,” “functional dyspepsia,” “non-ulcer dyspepsia,” “postprandial distress syndrome,” and “epigastric pain syndrome.” Boolean operators (AND, OR) were used to combine intervention and condition terms. No restrictions were placed on publication date. The full database search strategies are provided in Supplementary Material 2. Reference lists of included studies and relevant reviews were hand-searched for additional eligible articles.

### 2.4 Study selection

All retrieved records were exported to EndNote (Clarivate Analytics, Philadelphia, PA, USA) for automated deduplication. Records were then transferred to Zotero 5.0 (Corporation for Digital Scholarship, Vienna, VA, USA) for screening and full-text review. Two reviewers (XX and XQ) independently screened titles and abstracts against the eligibility criteria. Records considered potentially eligible by either reviewer advanced to full-text assessment, which was performed independently by the same two reviewers together with a third reviewer (JT). Disagreements at any stage were resolved through discussion; when consensus could not be reached, a senior reviewer (YR) adjudicated. The study selection process is summarized in the PRISMA 2020 flow diagram (Figure 1).

**FIGURE 1:**
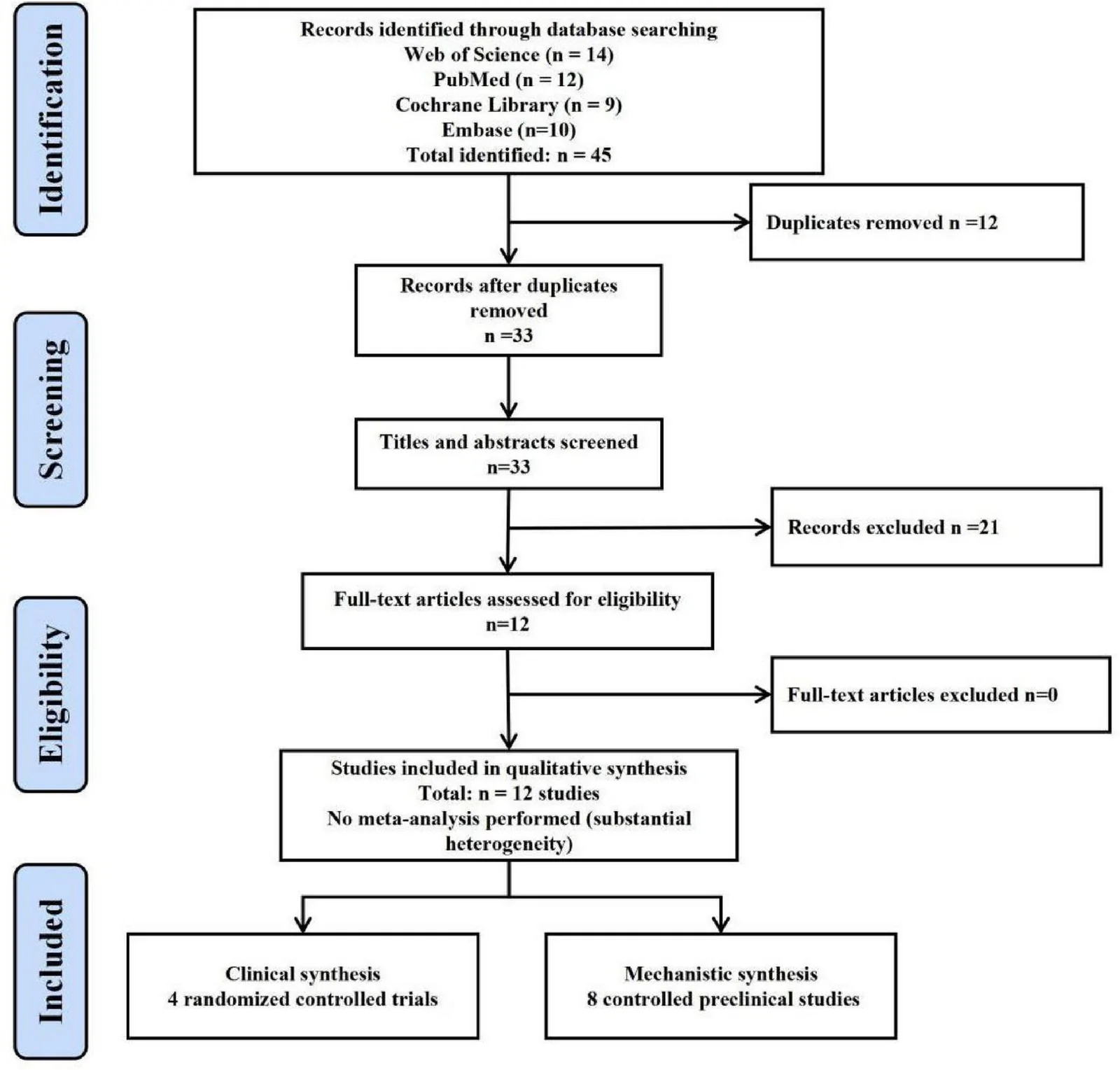
Flow chart of literature selection.

### 2.5 Data extraction

Data were extracted independently by two reviewers (JFS and YTR) using a standardized, pre-piloted extraction form. The extracted variables comprised: (1) bibliographic information (first author, year of publication, country); (2) study design and setting; (3) participant characteristics (sample size by arm, age, sex, diagnostic criteria, FD subtype); (4) intervention details (device, anatomical stimulation site, frequency, intensity, pulse width, session duration, treatment course, follow-up duration); (5) comparator details; (6) primary and secondary outcome measures; and (7) adverse events. For preclinical studies, additional data on animal model, FD induction method, and mechanistic biomarkers were extracted. When relevant outcome data were missing or unclear, the corresponding authors were contacted via email; if no response was received within 4 weeks, the available data were used. Discrepancies in extraction were resolved by discussion.

### 2.6 Risk of bias assessment

The risk of bias in the included RCTs was assessed independently by two reviewers (XX and JT) using the Cochrane Risk of Bias 2 (RoB 2) tool (Sterne et al., 2019), which evaluates five domains: (i) randomization process; (ii) deviations from intended interventions; (iii) missing outcome data; (iv) measurement of the outcome; and (v) selection of the reported result. Each domain was rated as low risk, some concerns, or high risk, and an overall judgment was derived. For controlled preclinical studies, the risk of bias was assessed using the SYRCLE risk of bias tool for animal studies (Hooijmans et al., 2014), which evaluates ten domains: sequence generation, baseline characteristics, allocation concealment, random housing, performance bias (blinding of caregivers and investigators), random outcome assessment, detection bias (blinding of outcome assessors), incomplete outcome data, selective outcome reporting, and other sources of bias. Disagreements were resolved by consensus or by a third reviewer (YR). Risk-of-bias judgments are summarized in Figure 2 (Cochrane RoB 2 for the included RCTs) and Figure 3 (SYRCLE for the controlled animal studies) and detailed in Supplementary Material 3.

**FIGURE 2:**
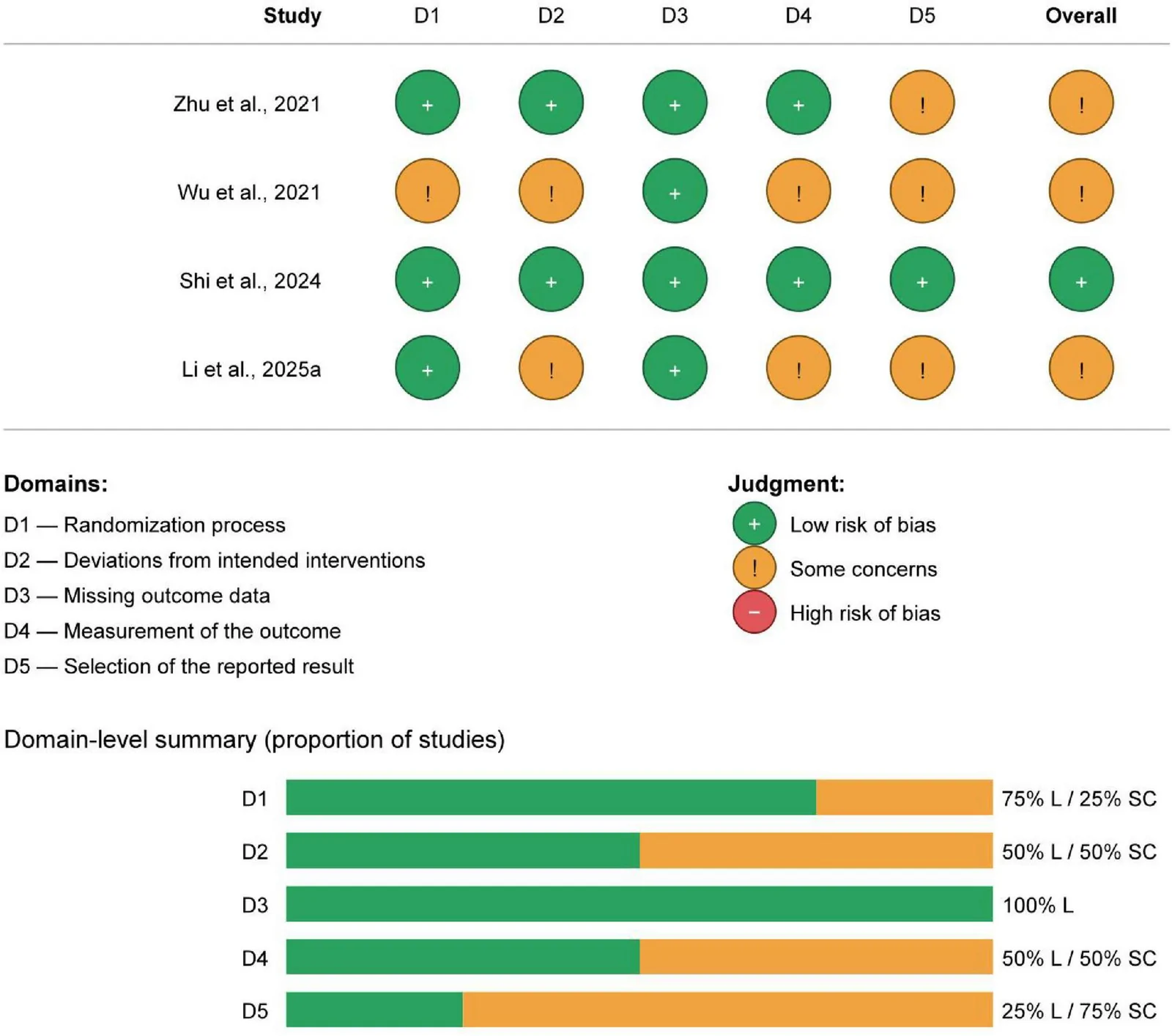
Risk-of-bias summary for the included randomized controlled trials, assessed with the Cochrane RoB 2 tool.

**FIGURE 3:**
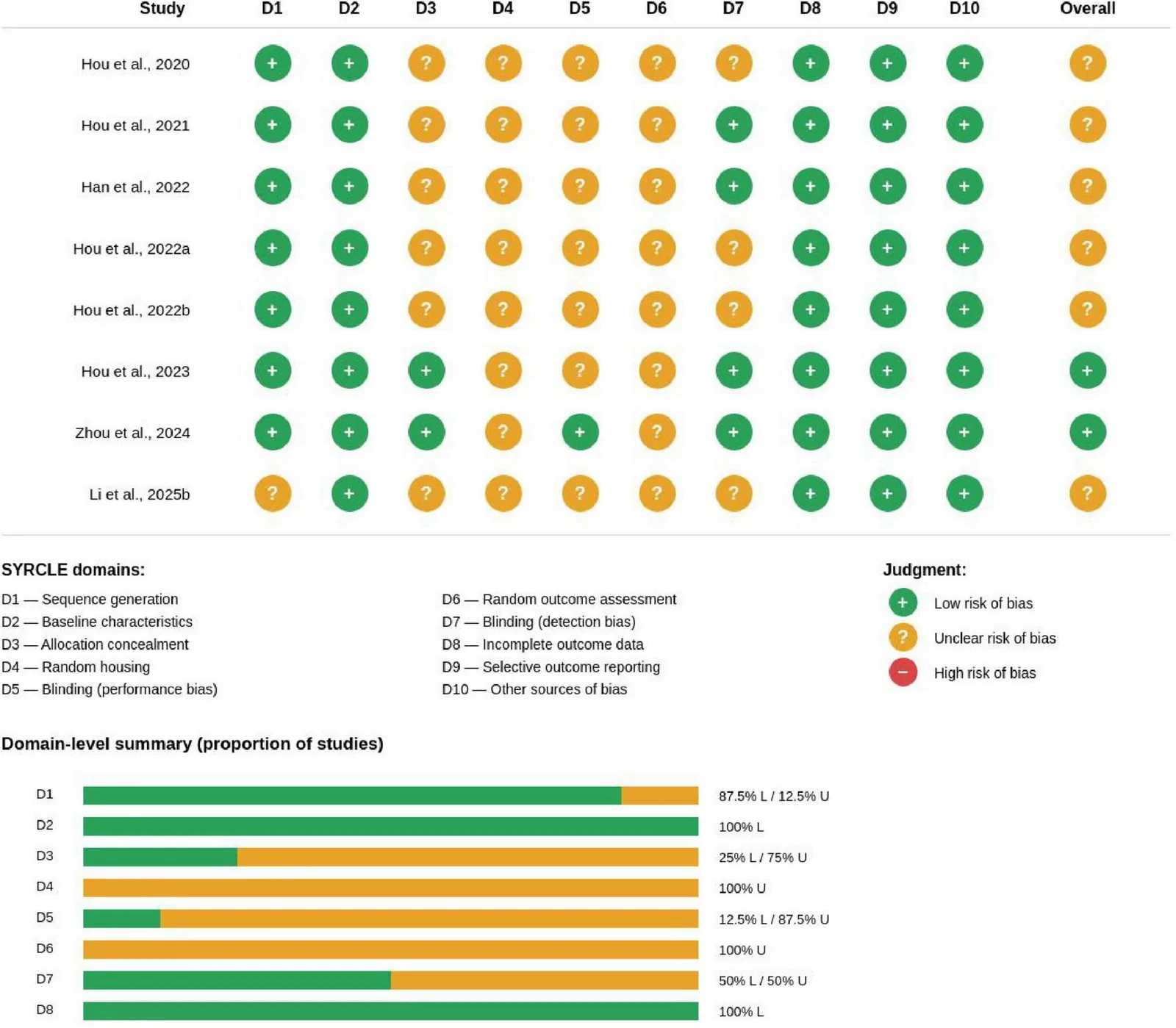
Risk-of-bias summary for the eight included controlled preclinical animal studies, assessed with the SYRCLE tool for animal studies. D1, sequence generation; D2, baseline characteristics; D3, allocation concealment; D4, random housing; D5, performance bias (blinding of personnel); D6, random outcome assessment; D7, detection bias (blinding of outcome assessors); D8, incomplete outcome data; D9, selective outcome reporting; D10, other sources of bias. Lower panel shows the domain-level proportion of studies judged at low (green) versus unclear (yellow) risk; no study was judged at high risk of bias on any domain.

### 2.7 Data synthesis

Given the small number of eligible clinical trials and substantial heterogeneity in stimulation parameters (frequency, intensity, treatment duration), choice of sham comparator, outcome instruments, and follow-up duration, quantitative meta-analysis was not appropriate and is, in any case, outside the scope of a scoping-review approach, which aims to map and characterize the evidence base rather than to derive aggregate effect estimates (Tricco et al., 2018; Peters et al., 2020). We therefore performed a structured narrative and tabular synthesis, presenting the included evidence in standardized data-charting tables (Tables 2, 3) and as a narrative description of the direction and consistency of effects (PRISMA-ScR Items 16–18). Studies were grouped by outcome domain (gastrointestinal symptoms, gastric physiological function, psychological and sleep outcomes, and safety) and by stimulation parameters. The direction and consistency of effects were summarized across studies, with particular attention to within-study comparisons against sham or active control. Preclinical mechanistic findings were synthesized thematically according to the five putative pathways linking taVNS to brain–gut axis modulation: autonomic and neurotransmitter regulation; cholinergic anti-inflammatory pathway and mucosal barrier repair; visceral hypersensitivity suppression; HPA-axis modulation; and brain–gut peptide regulation.

**TABLE 2:** Characteristics of clinical studies of taVNS in functional dyspepsia.

References	Country	Design	Population	Device/area	Stimulation parameters	Main findings
Zhu et al., 2021	China	RCT (double-blind, sham-controlled)	FD patients (*n* = 36)	Watch-sized stimulator (SNM-FDC01); taVNS: bilateral auricular cymba conchae; sham: lateral to wrist	25 Hz; 0.5 ms; 0.5–1.5 mA; 2 weeks	Improved gastric accommodation, increased proportion of normal gastric slow waves, enhanced vagal efferent activity, reduced dyspeptic symptoms, and lower anxiety/depression scores.
Wu et al., 2021	China	RCT	FD patients (*n* = 90)	Electronic acupuncture apparatus (Hwato, SDZ-IIB); taVNS: left cavum conchae; tnVNS: left scapha	30 Hz; 4–8 mA; 4 weeks	taVNS significantly reduced overall symptom scores, anxiety, and depression, and improved quality of life versus tnVNS. Effects were sustained at 4 and 12 weeks. A higher clinical effective rate was observed in the taVNS group.
Shi et al., 2024	China	Multicenter, randomized, double-blind, sham-controlled	FD patients (*n* = 300)	taVNS device (XD-Kerfun BS-VNS-001); V10 and V25 groups: left ear tragus; sham: left earlobe	V10: 10 Hz; V25: 25 Hz; 500 μs; 0.5–1.5 mA; 4 weeks	Both 10 Hz and 25 Hz significantly improved FD symptoms and quality of life, and reduced anxiety/depression versus sham. Effects were sustained at the 8-weeks follow-up. No significant difference was observed between 10 Hz and 25 Hz. Adverse events were mild and similar across groups.
Li J. et al., 2025	China	RCT (single-blind, sham-controlled)	FD patients with sleep disturbance (*n* = 54)	Watch-sized TEA device (SNM-FDC01); taVNS: bilateral cymba conchae; tnVNS: scapha	25 Hz; 0.5 ms; 2–10 mA; 4 weeks	Improved dyspeptic symptoms and sleep quality; enhanced FD-QoL; reduced anxiety/depression; increased gastric motility, gastric accommodation, serum melatonin, and vagal activity. Effects were sustained at the 4-weeks follow-up.

taVNS, transcutaneous auricular vagus nerve stimulation; tnVNS, transcutaneous non-auricular vagus nerve stimulation; RCT, randomized controlled trial; FD, functional dyspepsia; TEA, transcutaneous electrical acupoint stimulation; QoL, quality of life.

**TABLE 3:** Preclinical studies investigating the mechanism of taVNS in functional dyspepsia.

References	Rodent model	Device/area	taVNS parameters	Key biomarkers/ effects	Results and conclusion
Hou et al., 2020	Male SD rats	Modified Han’s acupoint-nerve-stimulator; taVNS: bilateral auricular concha cavity; sham: rim of auricular concha	Duration: 7 days; frequency/intensity not explicitly stated	Gastric sensitivity; serum MTL, CCK, GLP-1; serum IL-4, IL-10, IL-1β	Reduced gastric hypersensitivity; ↑MTL, ↓CCK, ↓GLP-1; ↑IL-4 and IL-10, ↓IL-1β. Peripheral anti-inflammatory effect and brain–gut peptide regulation.
Hou et al., 2021	Male SD rats	Transcutaneous electrical stimulator; taVNS: unilateral auricular concha	0.5 mA; 14 days	Gastric sensitivity; HRV; gastric antrum ACh; M3R expression	Reduced gastric hypersensitivity; ↑HF power and ↓LF/HF, indicating enhanced vagal activity; ↑ACh and M3R in the gastric antrum. Improved autonomic balance and vagal cholinergic activity.
Han et al., 2022	Male SD rats	Transcutaneous electroacupuncture stimulator; taVNS: unilateral auricular concha	100 Hz; 0.5 mA; 14 days	Gastric sensitivity; gastric emptying; duodenal ACh, α7nAChR, IL-6, IL-1β, TNF-α; NF-κB p65	Reduced gastric hypersensitivity; ↑gastric emptying; ↑ACh and α7nAChR in the duodenum; ↓NF-κB p65 and pro-inflammatory cytokines. Activation of the cholinergic anti-inflammatory pathway via α7nAChR/NF-κB.
Hou et al., 2022a	Male SD rats	Huatuo electroacupuncture device; taVNS: unilateral cymba/cavum conchae; sham: auricular helix	20/100 Hz; 2 mA; 0.2 ms; 14 days	General condition; gastric emptying; OFT; serum corticosterone, ACTH; hypothalamic CRF; hippocampal CRHR1	Improved general condition and gastric emptying; ↑exploratory behavior in the OFT; ↓corticosterone, ACTH, and hypothalamic CRF. Ameliorates FD and depressive-like behavior by inhibiting HPA-axis hyperactivity and modulating the CRF pathway.
Hou et al., 2022b	SPF SD rats	Hwato electroacupuncture apparatus; taVNS: cymba/cavum conchae; sham: rim of auricular concha	20/100 Hz; 2 mA; 14 days	ZO-1, occludin, β-catenin; eosinophil infiltration; gastric emptying; food intake	Improved general condition and gastric emptying; ↑ZO-1 and occludin in the duodenum; no change in β-catenin; minimal eosinophil infiltration. Repair of the duodenal mucosal barrier.
Hou et al., 2023	Male SD rats	Watch-sized stimulator (Ningbo Maida); taVNS: cymba/cavum conchae; sham: 0 mA	100 Hz; 0.5 mA; 14 days	Gastric emptying; OFT; vagal activity; serum TNF-α, IL-6, IL-1β, ACTH, corticosterone; duodenal NF-κB p65, ZO-1, occludin, β-catenin, DSG2; gastric ACh, M3R; hypothalamic CRF; amygdaloid CRF1	Reduced visceral hypersensitivity and improved gastric emptying; ↑vagal activity; ↓pro-inflammatory cytokines and duodenal NF-κB p65; restored mucosal integrity; suppressed HPA-axis hyperactivity. Effects were abolished by subdiaphragmatic vagotomy.
Zhou et al., 2024	Male SD rats	Microneedles + universal pulse generator; taVNS: bilateral cymba/cavum conchae; sham: bilateral earlobes	2 Hz; 0.7–1.0 mA; 5 days	Gastric hypersensitivity; NGF mRNA/protein in the gastric fundus; NGF, TrkA, PLC-γ, TRPV1 in NTS; TrkA/PLC-γ phosphorylation	↓AWR scores; ↓peripheral NGF in the gastric fundus; downregulated central NGF/TrkA/PLC-γ/TRPV1 in NTS; additive effect with the TrkA inhibitor K252a.
Li J. et al., 2025	SD rats	Device not specified; auricular vagus nerve region	Parameters not specified; 14 days	Gastric emptying; MTL; visceral sensitivity; TRPV1 in gastric antrum; duodenal ACh, α7nAChR, IL-6, IL-1β, TNF-α, NF-κB p65	Improved gastric emptying and ↑MTL; reduced visceral hypersensitivity; ↑duodenal ACh and α7nAChR; ↓pro-inflammatory cytokines and NF-κB p65. Effects were comparable to those of invasive SDVNS and were abolished by vagotomy.

SD, Sprague-Dawley; AWR, abdominal withdrawal reflex; HRV, heart rate variability; HF, high-frequency power; LF, low-frequency power; MTL, motilin; CCK, cholecystokinin; GLP-1, glucagon-like peptide 1; ACh, acetylcholine; α7nAChR, nicotinic acetylcholine receptor α7 subunit; M3R, muscarinic acetylcholine receptor M3; IL, interleukin; TNF-α, tumor necrosis factor α; NF-κB, nuclear factor κB; TRPV1, transient receptor potential vanilloid 1; NGF, nerve growth factor; TrkA, tropomyosin receptor kinase A; PLC-γ, phospholipase C-γ; HPA, hypothalamic–pituitary–adrenal; CRF, corticotropin-releasing factor; CRHR1, corticotropin-releasing hormone receptor 1; ACTH, adrenocorticotropic hormone; OFT, open field test; ZO-1, zonula occludens-1; DSG2, desmoglein 2; SDVNS, subdiaphragmatic vagus nerve stimulation; NTS, nucleus tractus solitarii. “↑” indicates an increase; “↓” indicates a decrease.

## 3 Results

### 3.1 Study selection

The systematic search identified records across the four databases, which were screened in two phases as described in Section “2.4 Study selection.” The flow of studies is depicted in Figure 1. Four RCTs (Zhu et al., 2021; Wu et al., 2021; Shi et al., 2024; Li J. et al., 2025) and eight controlled preclinical studies in rodents (Hou et al., 2020, 2021, Hou et al., 2022a,b, 2023; Han et al., 2022; Zhou et al., 2024; Li J. et al., 2025) met the eligibility criteria and were included in the synthesis.

### 3.2 Characteristics of included clinical studies

The characteristics of the four included clinical trials are summarized in Table 2. The trials enrolled a total of 480 participants with FD, with sample sizes ranging from 36 (Zhu et al., 2021) to 300 (Shi et al., 2024). All four were RCTs: three used a sham-controlled design in which sham stimulation was delivered to non-vagal sites such as the earlobe or auricular scapha to preserve blinding (Zhu et al., 2021; Shi et al., 2024; Li J. et al., 2025), and one used transcutaneous non-auricular vagus nerve stimulation (tnVNS) as an active comparator (Wu et al., 2021). Blinding strategies varied across studies, including double-blind, single-blind, and open-label designs. The stimulation frequency was set at 10 Hz, 25 Hz, or 30 Hz; the pulse width at 0.5 ms; and the current intensity at 0.5–1.5 mA in three studies and 2–10 mA in one (Li J. et al., 2025). The principal stimulation targets were the bilateral or unilateral cymba conchae, cavum conchae, or tragus. Treatment duration ranged from 2 to 4 weeks, with follow-up of 4–12 weeks.

### 3.3 Risk of bias in included studies

The risk-of-bias judgments for the four clinical trials are presented in Figure 2 and detailed in Supplementary Material 3. The overall risk of bias was low in two trials (Zhu et al., 2021; Shi et al., 2024) and raised some concerns in two trials (Wu et al., 2021; Li J. et al., 2025), primarily in the domains of blinding of participants and outcome assessors (single-blind designs) and selection of the reported result (no pre-registered protocol available). No trial was judged to be at high overall risk of bias. For the eight preclinical studies, the SYRCLE assessment (Figure 3) indicated overall low to unclear risk of bias, with the most common limitations being incomplete reporting of allocation concealment, random housing, and blinding of outcome assessors–patterns typical of animal studies in this field. Specifically, all eight studies were rated at low risk of bias for baseline characteristics, incomplete outcome data, selective outcome reporting, and other sources of bias, whereas random housing and random outcome assessment were rated as unclear in all studies and allocation concealment was rated as unclear in six of eight studies. None of the animal studies was judged to be at high risk of bias on any SYRCLE domain.

### 3.4 Synthesized clinical findings

#### 3.4.1 Effects on dyspeptic symptoms and gastric function

All four RCTs reported significant reductions in composite dyspeptic symptom scores with taVNS compared with sham or non-vagal control, and the effects were sustained throughout the follow-up periods documented in each trial (Zhu et al., 2021; Wu et al., 2021; Shi et al., 2024; Li J. et al., 2025). Beyond symptom relief, two trials specifically evaluated objective gastric physiological parameters and demonstrated enhanced gastric accommodation, an increased proportion of normal gastric slow waves on electrogastrography, and increased vagal efferent activity in the taVNS group (Zhu et al., 2021; Li J. et al., 2025). The largest trial–a multicenter, double-blind study of 300 patients (Shi et al., 2024) –provided the strongest evidence of symptomatic benefit and supported the durability of effects for up to 8 weeks after the end of treatment. The direction of effect was consistent across all four trials, despite differences in stimulation frequency, treatment duration, and choice of comparator.

#### 3.4.2 Effects on psychological comorbidities, sleep, and quality of life

Functional dyspepsia is frequently comorbid with anxiety, depression, and sleep disturbance, which contribute to symptom severity and to refractoriness to conventional therapy. All four trials measured psychological outcomes and reported significant reductions in anxiety and depression scores with taVNS relative to control (Zhu et al., 2021; Wu et al., 2021; Shi et al., 2024; Li J. et al., 2025). FD-related quality of life improved consistently across the three trials that measured it (Wu et al., 2021; Shi et al., 2024; Li J. et al., 2025). In FD patients with comorbid sleep disturbance, taVNS additionally improved sleep quality and was associated with an increase in serum melatonin (Li J. et al., 2025), indicating a coordinated effect on both gastrointestinal and psychobiological domains.

#### 3.4.3 Safety and adverse events

Adverse events were systematically reported only in the largest trial, in which taVNS at both 10 Hz and 25 Hz was associated with mild adverse events at rates comparable to sham (Shi et al., 2024). No serious adverse events were reported in any of the included trials, consistent with the broader literature on taVNS safety in other indications (Kim et al., 2022). Long-term safety beyond 12 weeks remains insufficiently characterized.

### 3.5 Synthesized preclinical mechanistic findings

The eight included preclinical studies (Table 3) consistently demonstrated that taVNS exerts beneficial effects in rodent models of FD through five interconnected mechanisms that collectively act on the brain–gut axis. A schematic summary of these five pathways is provided in Figure 4.

**FIGURE 4:**
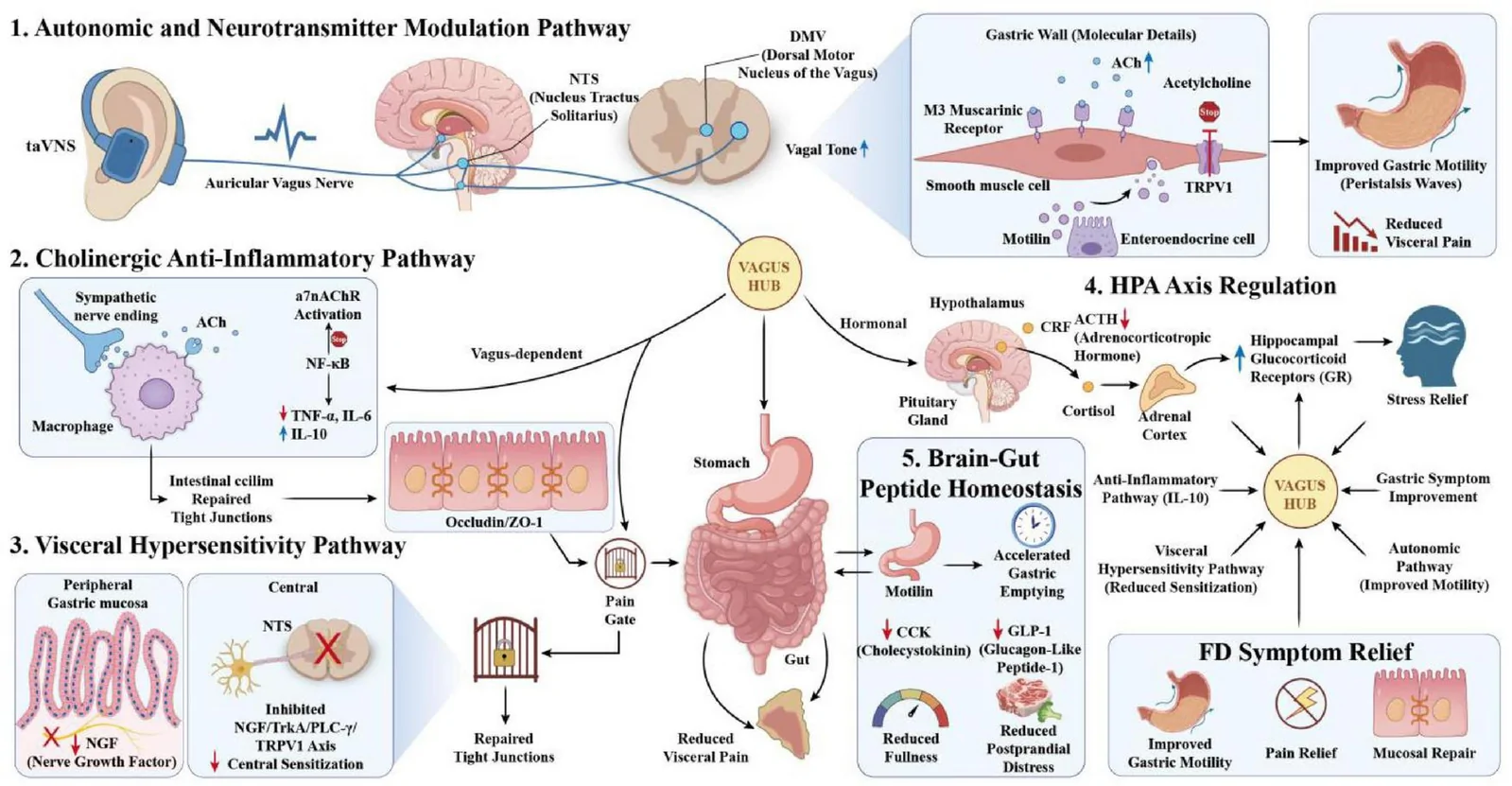
Schematic of the proposed multi-target mechanism by which taVNS modulates the brain–gut axis in functional dyspepsia. The five interconnected pathways act in concert: (i) regulation of autonomic balance and cholinergic neurotransmission; (ii) activation of the cholinergic anti-inflammatory pathway and repair of the duodenal mucosal barrier; (iii) suppression of visceral hypersensitivity via the peripheral and central NGF/TrkA/PLC-γ/TRPV1 axis; (iv) inhibition of HPA-axis hyperactivity; and (v) restoration of brain–gut peptide balance (motilin, cholecystokinin, GLP-1).

#### 3.5.1 Regulation of the autonomic nervous system and neurotransmitters

Autonomic dysfunction and associated neurotransmitter imbalances contribute to impaired gastric motility and visceral hypersensitivity in FD (Park et al., 2001; Oustamanolakis and Tack, 2012). In FD model rats, taVNS upregulated the expression of acetylcholine (ACh) and the nicotinic acetylcholine receptor α7 subunit (α7nAChR) in duodenal tissue, with downstream inhibition of NF-κB p65 signaling (Han et al., 2022). taVNS also improved autonomic balance, as reflected by increased high-frequency power and a decreased LF/HF ratio on heart rate variability analysis, in conjunction with increased ACh and M3R expression in the gastric antrum (Hou et al., 2021, 2023). In addition, taVNS activated the cholinergic anti-inflammatory pathway, promoted motilin release, and downregulated TRPV1 expression in the gastric antrum (Li J. et al., 2025).

#### 3.5.2 Cholinergic anti-inflammatory pathway and mucosal barrier repair

Low-grade duodenal immune activation and impaired mucosal barrier integrity are increasingly recognized as central to FD pathogenesis (Futagami and Wakabayashi, 2022; Gracie et al., 2019). In patients with FD, duodenal biopsies show mucosal eosinophil and mast cell hyperplasia together with reduced transepithelial electrical resistance, increased paracellular permeability, and altered expression of cell-to-cell adhesion proteins (Vanheel et al., 2014; Ceulemans et al., 2022), and a meta-analysis of 22 case–control studies confirmed higher duodenal eosinophil and mast cell counts, with a greater degranulated fraction, in FD than in controls (Shah et al., 2022). An intervention able to restrain this compartmentalized mucosal immune activation is therefore mechanistically attractive, and the cholinergic anti-inflammatory pathway (CAP) is the most fully characterized candidate.

Cholinergic anti-inflammatory pathway was originally described as an efferent vagal reflex in which brainstem outflow, relayed through the vagus and the splenic nerve, elicits acetylcholine (ACh) release from a specialized population of choline acetyltransferase-expressing T cells in the spleen; ACh then engages the α7 nicotinic acetylcholine receptor (α7nAChR) on cytokine-producing macrophages and suppresses NF-κB-dependent transcription of TNF-α, IL-1β, and IL-6 (Tracey, 2002; Wang et al., 2003). Receptor ligation activates JAK2/STAT3 signaling and prevents nuclear translocation of NF-κB p65, attenuating pro-inflammatory transcription without abolishing host defense (de Jonge et al., 2005). In the gut wall, however, the effector arm is anatomically distinct and spleen-independent: vagal efferents do not innervate intestinal macrophages directly but terminate on cholinergic myenteric neurons lying in close apposition to α7nAChR-expressing resident macrophages, which transduce the signal locally (Matteoli and Boeckxstaens, 2013; Cailotto et al., 2014; Matteoli et al., 2014). Because taVNS is thought to act predominantly through afferent activation of the NTS with secondary recruitment of dorsal motor nucleus output, it is this vago-vagal enteric loop, rather than the vago-splenic loop, that is most plausibly engaged in the upper gut.

The preclinical FD literature is consistent with this scheme. In rodent FD models, taVNS increased ACh and α7nAChR expression in duodenal tissue, reduced NF-κB p65, and lowered IL-6, IL-1β, and TNF-α (Han et al., 2022; Li J. et al., 2025), while the anti-inflammatory cytokines IL-4 and IL-10 rose (Hou et al., 2020). In parallel, the tight-junction proteins ZO-1 and occludin were upregulated and mucosal permeability fell, whereas β-catenin was unchanged and eosinophil infiltration remained minimal (Hou et al., 2022b) – a dissociation consistent with an indirect barrier effect operating through suppression of inflammation-induced tight-junction degradation rather than a direct action on adherens junctions (Puthanmadhom Narayanan et al., 2022). Two studies reported that subdiaphragmatic vagotomy abolished both the anti-inflammatory and the barrier effects (Hou et al., 2023; Li J. et al., 2025), establishing dependence on an intact vagal trunk. It should be noted, however, that subdiaphragmatic vagotomy severs afferent and efferent fibers simultaneously; these experiments therefore demonstrate vagal dependence but do not isolate the efferent cholinergic arm from afferent-driven central mechanisms such as HPA-axis modulation (Section “3.5.4 Modulation of the HPA axis and stress response”). No FD study has yet used selective α7nAChR antagonism or receptor-knockout animals to establish that the receptor is required, so the causal chain from taVNS to α7nAChR engagement remains inferred from correlated expression changes.

Direct confirmation of CAP activation in humans with FD is, at present, absent. None of the four included clinical trials measured any inflammatory or barrier endpoint: no trial reported circulating or mucosal cytokines, duodenal histology, mucosal permeability, or α7nAChR expression (Zhu et al., 2021; Wu et al., 2021; Shi et al., 2024; Li J. et al., 2025). The inference that symptomatic benefit in patients is mediated by CAP is therefore extrapolated from rodents rather than demonstrated. The closest available human data come from adjacent conditions and are not uniform in direction. In constipation-predominant IBS, 4 weeks of taVNS reduced serum TNF-α and IL-6 relative to sham alongside symptomatic improvement and enhanced vagal activity (Shi et al., 2021); in small uncontrolled pilot studies of invasive cervical VNS in Crohn’s disease, C-reactive protein and fecal calprotectin fell in most patients over 6–12 months (Bonaz et al., 2016; Sinniger et al., 2020). Conversely, in a randomized study of experimental human endotoxemia, transvenous VNS did not attenuate the plasma cytokine response, *ex vivo* leukocyte cytokine production, or neutrophil phagocytosis (Kox et al., 2015), indicating that the anti-inflammatory reflex demonstrated so robustly in rodents is not uniformly reproducible in humans and may be contingent on stimulation route, parameters, and inflammatory context.

Three further considerations temper translation of CAP to FD specifically. First, the effector cell populations differ: the rodent CAP literature centers on macrophages, whereas human FD duodenal immune activation is dominated by an eosinophil–mast cell axis (Ceulemans et al., 2022; Shah et al., 2022), whose responsiveness to α7nAChR ligation is less well established. Second, circulating cytokines – the only inflammatory readout so far used in human neuromodulation trials in disorders of gut–brain interaction – are an imperfect surrogate for the localized, low-grade duodenal immune activation that characterizes FD, and unchanged systemic markers would not exclude a mucosal effect. Third, the preclinical CAP experiments used stimulation frequencies of 20–100 Hz (Han et al., 2022; Hou et al., 2022a,b, 2023), whereas the clinical trials used 10–30 Hz (Zhu et al., 2021; Wu et al., 2021; Shi et al., 2024; Li J. et al., 2025); whether the frequencies that produce clinical benefit are also those that engage the anti-inflammatory reflex is unknown, and no dose–response data for the anti-inflammatory effect of taVNS exist in humans. CAP should therefore be regarded as the best-supported candidate mechanism for an anti-inflammatory action of taVNS in FD, and not as an established mediator of its clinical efficacy.

#### 3.5.3 Suppression of visceral hypersensitivity

Visceral hypersensitivity is a key pathophysiological feature of FD (Keohane and Quigley, 2006; Zhao et al., 2020). In gastric-hypersensitive FD model rats, taVNS reduced abdominal withdrawal reflex scores in response to gastric distension by downregulating peripheral NGF expression in the gastric fundus and inhibiting central NGF/TrkA/PLC-γ signaling in the nucleus of the solitary tract, with reduced phosphorylation of TrkA and PLC-γ and reduced TRPV1 expression (Zhou et al., 2024). Activation of the cholinergic anti-inflammatory pathway also decreased TRPV1 expression in the gastric antrum and reduced the electromyographic response of the trapezius to gastric distension (Li J. et al., 2025). Together, these data indicate that taVNS modulates both peripheral and central nodes of the visceral nociceptive network.

#### 3.5.4 Modulation of the HPA axis and stress response

Hyperactivation of the HPA axis is closely linked to FD with depressive-like behavior (Mutsuura et al., 2009; Nardone and Compare, 2014). taVNS reduced elevated hypothalamic CRF, serum ACTH, and corticosterone levels in FD model rats and upregulated hippocampal CRHR1 expression, thereby restoring negative-feedback regulation of the HPA axis (Hou et al., 2022a). These changes were accompanied by improvements in depressive-like behaviors, including increased sucrose preference and reduced immobility in the forced swim test (Hou et al., 2023), supporting a coordinated effect on gastrointestinal and affective symptoms.

#### 3.5.5 Regulation of brain-gut peptides

Brain–gut peptides link central nervous system function to gastrointestinal motility, secretion, and sensation, and their imbalance contributes to delayed gastric emptying and visceral hypersensitivity in FD (Mengting et al., 2024; Xue et al., 2019; Chua and Keeling, 2006; Tu et al., 2022). In FD model rats with decreased serum motilin and increased CCK and GLP-1, taVNS rebalanced these peptides–restoring motilin and reducing CCK and GLP-1 toward control levels (Hou et al., 2020) –and increased gastric emptying with reduced gastric residue (Li J. et al., 2025). Restoration of the brain–gut peptide network thereby contributes to the relief of postprandial fullness and early satiety.

## 4 Discussion

### 4.1 Summary of main findings

This scoping review of four RCTs (*n* = 480 participants) and eight controlled preclinical studies provides the first structured evidence map of the clinical and mechanistic literature on taVNS for FD. The clinical evidence is consistent in direction: across studies spanning sample sizes from 36 to 300, stimulation frequencies of 10–30 Hz, and treatment durations of 2–4 weeks, taVNS produced significant improvements in dyspeptic symptom scores; in comorbid anxiety, depression, and sleep disturbance; and in FD-related quality of life relative to sham or non-vagal comparators, with effects sustained for up to 12 weeks of follow-up. Objective gastric physiological measures–gastric accommodation, gastric slow-wave normalization, and vagal efferent activity indices–improved in the trials that measured them. Adverse events were mild and comparable to those observed with sham stimulation. The preclinical evidence is mechanistically coherent and points to five interconnected pathways: autonomic and cholinergic neurotransmitter regulation; activation of the cholinergic anti-inflammatory pathway with duodenal mucosal barrier repair; suppression of visceral hypersensitivity through the NGF/TrkA/PLC-γ/TRPV1 axis; HPA-axis modulation; and rebalancing of brain–gut peptides. Critically, vagotomy abolishes the central and peripheral effects of taVNS in rodent models (Hou et al., 2023; Li J. et al., 2025), supporting causal involvement of vagal afferent signaling rather than non-specific cutaneous effects. These mechanistic conclusions, however, derive entirely from rodent experiments. No clinical trial in FD has yet measured an inflammatory, barrier, or brain–gut peptide endpoint, and the five pathways summarized here are therefore candidate rather than confirmed mediators of the effects observed in patients.

### 4.2 Comparison with the existing literature

Earlier reviews of taVNS for FD have been narrative in design and have not applied transparent eligibility criteria or formal risk-of-bias appraisal, limiting the strength of inferences that can be drawn. The systematic synthesis presented here confirms and extends previous narrative observations by quantifying the small but consistent clinical evidence base, appraising the methodological quality of each trial against the Cochrane RoB 2 framework, and mapping the preclinical mechanistic literature onto an integrated brain–gut axis schema. The convergence between clinical effects (symptom relief, improved vagal indices, and reduced anxiety and depression) and preclinical mechanisms (vagal activation, cholinergic anti-inflammatory signaling, and HPA-axis normalization) supports the biological plausibility of the observed clinical benefits. Positioned against the wider DGBI literature, the FD evidence base is more mature than that for gastroparesis, in which non-invasive vagal stimulation has so far been assessed only in proof-of-concept and uncontrolled open-label designs (Paulon et al., 2017; Gottfried-Blackmore et al., 2020), yet less mature than that for IBS, in which sham-controlled auricular neurostimulation has been replicated by independent groups in different countries (Kovacic et al., 2017; Krasaelap et al., 2020; Shi et al., 2021; Liu C. et al., 2025). The convergent direction of effect across these three conditions, together with the shared mechanistic substrate of vagal afferent signaling, supports a common brain–gut mode of action; the differences in replication status explain why the inferences that can currently be drawn for FD remain more provisional than those for IBS.

Placed alongside other neuromodulation strategies for gastroduodenal symptoms, taVNS occupies a favorable position on the invasiveness–evidence trade-off, although no head-to-head comparison has been performed. Transcutaneous electrical acustimulation and electroacupuncture at ST36 or PC6 improve dyspeptic symptoms and gastric motility through partly overlapping vagal and autonomic mechanisms and rest on a larger, though methodologically heterogeneous, trial literature (Guo et al., 2020); taVNS differs mainly in targeting a defined cutaneous vagal field rather than a meridian-based acupoint. Invasive cervical VNS has never been tested in FD, and its gastroenterological use is confined to small uncontrolled Crohn’s disease series (Bonaz et al., 2016; Sinniger et al., 2020), at the cost of surgical implantation and stimulation-related dysphonia. Implanted gastric electrical stimulation is licensed only for refractory gastroparesis, where a large randomized crossover trial reduced vomiting frequency without improving gastric emptying or quality of life (Ducrotte et al., 2020) – a reminder that device-based effects on upper-gut symptoms need not be motility-mediated. Central neuromodulators, principally tricyclic antidepressants, remain the best-established pharmacological option for refractory FD but are limited by anticholinergic and sedative effects (Drossman et al., 2018). Against this background, the appeal of taVNS lies in being non-invasive, self-administrable, and free of systemic drug exposure; its principal disadvantage relative to these comparators is the small size and single-country provenance of its evidence base.

### 4.3 Implications for clinical practice

Transcutaneous auricular vagus nerve stimulation offers a non-invasive, well-tolerated, and mechanistically multi-target option for patients with FD, particularly those with comorbid anxiety, depression, or sleep disturbance–a subgroup that is poorly served by current pharmacotherapy. Available trials suggest that 25 Hz stimulation, a pulse width of 0.5 ms, an intensity within individual tolerance, and a 4-weeks treatment course produce clinically meaningful symptom relief that persists for at least 8 weeks after the end of treatment (Shi et al., 2024; Li J. et al., 2025). However, given the small number of trials and the absence of head-to-head comparisons with active pharmacological controls, taVNS should at present be regarded as a promising adjunct rather than a replacement for established therapy. Routine clinical adoption should await confirmation in adequately powered, geographically diverse trials with standardized devices and parameters.

### 4.4 Limitations

Several limitations should be considered when interpreting these findings. First, only four RCTs met the eligibility criteria, three of which had sample sizes below 100; the synthesis is therefore inevitably constrained by limited statistical power and by potential geographic bias, as all included trials were conducted in China. No independent replication outside China is currently available, and external validity and reproducibility across ethnic groups, health-care systems, dietary contexts, and devices therefore remain unestablished–a constraint that distinguishes the FD literature from the multinational evidence base now available for auricular neurostimulation in IBS. Second, substantial heterogeneity in stimulation parameters (frequency, intensity, and duration), in the choice of sham comparator, in blinding strategy, and in outcome instruments precluded quantitative meta-analysis, which is in any case beyond the remit of a scoping review, and the narrative synthesis cannot generate an aggregate effect estimate or detect publication bias through funnel-plot methods. Third, follow-up periods in the included trials did not exceed 12 weeks, leaving the long-term efficacy, durability, and safety of taVNS undetermined. Fourth, the mechanistic synthesis relies entirely on rodent studies. This is particularly consequential for the cholinergic anti-inflammatory pathway, the most fully developed of the five proposed mechanisms, which has never been interrogated in patients with FD: none of the four included trials measured circulating or mucosal cytokines, duodenal histology, mucosal permeability, or α7nAChR expression. Supportive human data exist only for adjacent conditions and are inconsistent – reductions in circulating pro-inflammatory cytokines after taVNS in IBS-C (Shi et al., 2021) and in C-reactive protein and fecal calprotectin after invasive VNS in uncontrolled Crohn’s disease pilots (Bonaz et al., 2016; Sinniger et al., 2020), but no attenuation of the cytokine response to endotoxin in a randomized human study (Kox et al., 2015). Direct mechanistic validation in humans, combining functional MRI, HRV, and mucosal as well as systemic inflammatory readouts, is therefore required before CAP can be regarded as a mechanism of clinical benefit in FD rather than a biologically plausible hypothesis. Fifth, a contribution of placebo mechanisms to the observed benefit cannot be excluded. FD endpoints are subjective, and the pooled placebo response rate in pharmacological FD trials is approximately 39% (Bosman et al., 2023); sham devices may elicit larger placebo effects than inert pills (Kaptchuk et al., 2006), and taVNS combines a visible device, a therapeutic ritual, and repeated clinician contact. Although all four trials used a sham or non-vagal comparator and reported between-group differences, two were single-blind or open-label and none reported blinding success or sham credibility, so the stimulation-specific component of the effect remains incompletely separated from expectation. Sixth, symptom assessment was not uniform: only two trials used an FD-specific validated instrument – the modified FD Symptom Diary (Shi et al., 2024) and the Nepean Dyspepsia Index (Li J. et al., 2025) – whereas the other two relied on non-standardized composite symptom scores (Zhu et al., 2021; Wu et al., 2021), with correspondingly different responder definitions; psychological and sleep outcomes were captured with validated but generic scales. This limits cross-trial comparability and precludes judging whether the reported changes exceed a minimal clinically important difference. Seventh, this review did not include unpublished material, conference abstracts, or non-English-language studies, which may introduce language and publication bias. Finally, the protocol of this scoping review was not prospectively registered on the Open Science Framework (OSF); although PROSPERO does not currently accept scoping-review protocols, prospective registration on a generic platform such as OSF would have further enhanced methodological transparency and is recommended for future updates of this evidence map.

### 4.5 Implications for future research

#### 4.5.1 Clinical trial design

Future trials should be adequately powered, multicenter, and geographically diverse, with prospectively registered protocols. Standardization of stimulation parameters–including head-to-head comparisons of frequency (e.g., 10 Hz versus 25 Hz), intensity, and treatment duration–is needed to define the optimal dose. Follow-up periods of at least 6–12 months are required to characterize the durability of effect and long-term safety, with systematic monitoring for adverse events affecting auricular skin, auditory function, and autonomic homeostasis. Pragmatic trials comparing taVNS with active pharmacological controls and combination regimens (e.g., taVNS plus prokinetics, probiotics, or cognitive behavioral therapy) would inform the real-world positioning of the technique. Trials should additionally adopt FD-specific validated instruments with pre-specified responder definitions, and incorporate a placebo run-in together with formal assessment of blinding success and sham credibility, so that the stimulation-specific component of the response can be quantified.

#### 4.5.2 Precision medicine and patient stratification

Multimodal biomarker panels–incorporating gut microbiota composition, brain functional connectivity, autonomic indices, and inflammatory cytokine profiles–could be developed into predictive models to identify FD patients most likely to respond to taVNS. Subgroup analyses by Rome IV subtype (PDS versus EPS), comorbid anxiety/depression, sleep disturbance, age, sex, and baseline autonomic function would refine treatment indications.

#### 4.5.3 Mechanistic validation in humans

Although the preclinical mechanistic evidence is internally coherent, the relative contributions of the five proposed pathways to clinical efficacy remain unclear. Future studies should combine concurrent measurements of vagal activity (HRV), central modulation (functional MRI of the NTS, locus coeruleus, hypothalamus, and amygdala), peripheral inflammation, brain–gut peptide levels, and gut microbiota in humans receiving taVNS, ideally with sham-controlled crossover designs to dissociate stimulation-specific from non-specific effects. For the cholinergic anti-inflammatory pathway specifically, a sham-controlled trial incorporating paired pre- and post-treatment duodenal biopsies would be both informative and technically feasible, since such endpoints are already used in interventional FD studies. Quantification of eosinophil and mast cell counts and degranulation, mucosal cytokine transcripts, α7nAChR expression, and tight-junction protein expression, together with Ussing-chamber measurement of transepithelial electrical resistance and paracellular permeability, would test the mucosal predictions of the rodent work directly, while paired serum cytokines and HRV would establish whether any mucosal change tracks the increase in vagal efferent activity. Formally relating the magnitude of biomarker change to symptom response would further indicate whether CAP engagement mediates clinical benefit or is a parallel epiphenomenon.

#### 4.5.4 Standardization of devices and protocols

Variability in electrode design, ear-fixation stability, and waveform delivery limits cross-study comparability and clinical translation. Consensus on minimum reporting standards for taVNS protocols (in line with the CONSORT-NPT extension for non-pharmacological treatments), validation of portable home-use devices, and development of clinical practice guidelines for taVNS in FD are needed to support broader implementation.

## 5 Conclusion

This review characterizes taVNS as a safe, well-tolerated, and mechanistically plausible non-invasive neuromodulation for FD acting on multiple brain–gut axis nodes. However, given the small number of trials, parameter heterogeneity, and limited follow-up, taVNS currently represents a promising investigational approach with encouraging preliminary evidence, rather than an established therapeutic option. Adequately powered, multicenter RCTs with standardized protocols and long-term follow-up are needed to confirm efficacy and define optimal regimens. Direct evidence linking the proposed brain–gut mechanisms to symptom improvement in patients is currently lacking, and the contribution of placebo mechanisms to the reported benefit remains to be quantified.

## Data Availability

The original contributions presented in this study are included in this article/Supplementary material, further inquiries can be directed to the corresponding authors.
