# Supplementary material for: Transcutaneous auricular vagus nerve stimulation targets the brain-gut axis to treat functional dyspepsia: a scoping review of clinical evidence and mechanistic research
Source: Front Neurosci. 2026 Sep 16;20:1876591. doi: 10.3389/fnins.2026.1876591 (PMC13623837; doi:10.3389/fnins.2026.1876591)
Supplement: Supplementary file 1 [file Table_1.docx]

**Supplementary Material S1**

**PRISMA 2020 Checklist**

*Transcutaneous auricular vagus nerve stimulation targets the brain–gut axis to treat functional dyspepsia: a systematic review of clinical evidence and mechanistic research*

*This checklist follows the PRISMA 2020 statement (Page MJ, McKenzie JE, Bossuyt PM, et al. The PRISMA 2020 statement: an updated guideline for reporting systematic reviews.* ***BMJ*** *2021;372:n71). Section and item numbers correspond to the 27-item PRISMA 2020 checklist. "Location" refers to the section or page in the manuscript where each item is reported.*

| **Item #** | **Section / topic** | **Checklist item** | **Reported in (manuscript section)** |
| --- | --- | --- | --- |
| **TITLE** | | | |
| **1** | **Title** | Identify the report as a systematic review. | Title ("... A Systematic Review of Clinical Evidence and Mechanistic Research") |
| **ABSTRACT** | | | |
| **2** | **Abstract** | See the PRISMA 2020 for Abstracts checklist. | Abstract — structured as Background, Methods, Results, Conclusion |
| **INTRODUCTION** | | | |
| **3** | **Rationale** | Describe the rationale for the review in the context of existing knowledge. | Section 1 (Introduction), paragraphs 1–4 |
| **4** | **Objectives** | Provide an explicit statement of the objective(s) or question(s) the review addresses. | Section 1 (Introduction), final paragraph |
| **METHODS** | | | |
| **5** | **Eligibility criteria** | Specify the inclusion and exclusion criteria for the review and how studies were grouped for the syntheses. | Section 2.2 (Eligibility criteria); Table 1 (PICOS framework) |
| **6** | **Information sources** | Specify all databases, registers, websites, organisations, reference lists, and other sources searched or consulted to identify studies. Specify the date when each source was last searched or consulted. | Section 2.3 (Information sources and search strategy) |
| **7** | **Search strategy** | Present the full search strategies for all databases, registers, and websites, including any filters and limits used. | Section 2.3; Supplementary Material S2 (full search strategies) |
| **8** | **Selection process** | Specify the methods used to decide whether a study met the inclusion criteria of the review, including how many reviewers screened each record and each report retrieved, whether they worked independently, and, if applicable, details of automation tools used in the process. | Section 2.4 (Study selection) |
| **9** | **Data collection process** | Specify the methods used to collect data from reports, including how many reviewers collected data from each report, whether they worked independently, any processes for obtaining or confirming data from study investigators, and, if applicable, details of automation tools used. | Section 2.5 (Data extraction) |
| **10a** | **Data items — outcomes** | List and define all outcomes for which data were sought. Specify whether all results compatible with each outcome domain in each study were sought (e.g. for all measures, time points, analyses), and if not, the methods used to decide which results to collect. | Section 2.5; Table 1 (Outcomes); Section 2.1 (PICOS) |
| **10b** | **Data items — other variables** | List and define all other variables for which data were sought (e.g. participant and intervention characteristics, funding sources). Describe any assumptions made about any missing or unclear information. | Section 2.5 (Data extraction) |
| **11** | **Study risk of bias assessment** | Specify the methods used to assess risk of bias in the included studies, including details of the tool(s) used, how many reviewers assessed each study and whether they worked independently, and, if applicable, details of automation tools used. | Section 2.6 (Risk of bias assessment); Supplementary Material S3 |
| **12** | **Effect measures** | Specify for each outcome the effect measure(s) (e.g. risk ratio, mean difference) used in the synthesis or presentation of results. | Section 2.7 (Data synthesis) — narrative effect direction and consistency; no quantitative effect measure used due to heterogeneity |
| **13a** | **Synthesis methods — eligibility for syntheses** | Describe the processes used to decide which studies were eligible for each synthesis. | Section 2.7 (Data synthesis) |
| **13b** | **Synthesis methods — data preparation** | Describe any methods required to prepare the data for presentation or synthesis, such as handling of missing summary statistics, or data conversions. | Section 2.5 (Data extraction); Section 2.7 |
| **13c** | **Synthesis methods — tabulation/visual displays** | Describe any methods used to tabulate or visually display results of individual studies and syntheses. | Section 2.7; Tables 2 and 3; Figure 2 (RoB summary) |
| **13d** | **Synthesis methods — quantitative synthesis** | Describe any methods used to synthesize results and provide a rationale for the choice(s). If meta-analysis was performed, describe the model(s), method(s) to identify the presence and extent of statistical heterogeneity, and software package(s) used. | Section 2.7 — narrative synthesis performed in accordance with the SWiM guideline (Campbell et al., 2020); meta-analysis not performed due to heterogeneity |
| **13e** | **Synthesis methods — heterogeneity** | Describe any methods used to explore possible causes of heterogeneity among study results (e.g. subgroup analysis, meta-regression). | Section 2.7 — grouping by outcome domain and stimulation parameters; further discussed in Section 4.4 (Limitations) |
| **13f** | **Synthesis methods — sensitivity** | Describe any sensitivity analyses conducted to assess robustness of the synthesized results. | Not applicable — quantitative synthesis not performed |
| **14** | **Reporting bias assessment** | Describe any methods used to assess risk of bias due to missing results in a synthesis (arising from reporting biases). | Not applicable — quantitative synthesis not performed; funnel-plot methods could not be applied (noted in Section 4.4 Limitations) |
| **15** | **Certainty assessment** | Describe any methods used to assess certainty (or confidence) in the body of evidence for an outcome. | Risk of bias assessed for each included study (Section 2.6; Figure 2; Supplementary Material S3); formal GRADE assessment not performed |
| **RESULTS** | | | |
| **16a** | **Study selection — flow** | Describe the results of the search and selection process, from the number of records identified in the search to the number of studies included in the review, ideally using a flow diagram. | Section 3.1 (Study selection); Figure 1 (PRISMA 2020 flow diagram) |
| **16b** | **Study selection — excluded** | Cite studies that might appear to meet the inclusion criteria, but which were excluded, and explain why they were excluded. | Figure 1 (counts and reasons by category) |
| **17** | **Study characteristics** | Cite each included study and present its characteristics. | Section 3.2 (Characteristics of included studies); Table 2 (clinical); Table 3 (preclinical) |
| **18** | **Risk of bias in studies** | Present assessments of risk of bias for each included study. | Section 3.3 (Risk of bias); Figure 2; Supplementary Material S3 |
| **19** | **Results of individual studies** | For all outcomes, present, for each study: (a) summary statistics for each group (where appropriate) and (b) an effect estimate and its precision (e.g. confidence/credible interval), ideally using structured tables or plots. | Tables 2 and 3; Sections 3.4–3.5 (narrative synthesis with study-level findings) |
| **20a** | **Results of syntheses — characteristics** | For each synthesis, briefly summarise the characteristics and risk of bias among contributing studies. | Sections 3.2–3.3 |
| **20b** | **Results of syntheses — results** | Present results of all statistical syntheses conducted. If meta-analysis was done, present for each the summary estimate and its precision (e.g. confidence/credible interval) and measures of statistical heterogeneity. If comparing groups, describe the direction of the effect. | Sections 3.4 (clinical) and 3.5 (mechanistic) — narrative synthesis describing direction and consistency of effects |
| **20c** | **Results of syntheses — heterogeneity** | Present results of all investigations of possible causes of heterogeneity among study results. | Section 4.4 (Limitations) — heterogeneity in stimulation parameters, comparators, outcome instruments, follow-up duration |
| **20d** | **Results of syntheses — sensitivity** | Present results of all sensitivity analyses conducted to assess the robustness of the synthesized results. | Not applicable |
| **21** | **Reporting biases** | Present assessments of risk of bias due to missing results (arising from reporting biases) for each synthesis assessed. | Section 4.4 (Limitations) — small number of studies precludes formal reporting-bias assessment |
| **22** | **Certainty of evidence** | Present assessments of certainty (or confidence) in the body of evidence for each outcome assessed. | Section 3.3 and Section 4.4 — RoB judgments and narrative description of evidence strength |
| **DISCUSSION** | | | |
| **23a** | **Discussion — interpretation in context of other evidence** | Provide a general interpretation of the results in the context of other evidence. | Section 4.1 (Summary of main findings); Section 4.2 (Comparison with existing literature) |
| **23b** | **Discussion — limitations of evidence** | Discuss any limitations of the evidence included in the review. | Section 4.4 (Limitations) |
| **23c** | **Discussion — limitations of review processes** | Discuss any limitations of the review processes used. | Section 4.4 (Limitations) — retrospective registration noted; English-only inclusion noted |
| **23d** | **Discussion — implications** | Discuss implications of the results for practice, policy, and future research. | Section 4.3 (Implications for clinical practice); Section 4.5 (Implications for future research) |
| **OTHER INFORMATION** | | | |
| **24a** | **Registration and protocol — registration** | Provide registration information for the review, including register name and registration number, or state that the review was not registered. | Section 2 Methods, "Protocol registration" paragraph — not registered |
| **24b** | **Registration and protocol — protocol** | Indicate where the review protocol can be accessed, or state that a protocol was not prepared. | Section 2 Methods, "Protocol registration" paragraph — protocol not separately registered; full methods reported in Section 2 and Supplementary Materials S1–S3 |
| **24c** | **Registration and protocol — amendments** | Describe and explain any amendments to information provided at registration or in the protocol. | Not applicable — no registered protocol |
| **25** | **Support** | Describe sources of financial or non-financial support for the review, and the role of the funders or sponsors in the review. | Declarations — Funding ("... Beijing Administration of Traditional Chinese Medicine, Project No. BJZYYB202535; the funder had no role in...") |
| **26** | **Competing interests** | Declare any competing interests of review authors. | Declarations — Conflicts of interest |
| **27** | **Availability of data, code, and other materials** | Report which of the following are publicly available and where they can be found: template data collection forms; data extracted from included studies; data used for all analyses; analytic code; any other materials used in the review. | Declarations — Data availability; Supplementary Materials S1–S3 |

# **Reference**

Page MJ, McKenzie JE, Bossuyt PM, Boutron I, Hoffmann TC, Mulrow CD, Shamseer L, Tetzlaff JM, Akl EA, Brennan SE, Chou R, Glanville J, Grimshaw JM, Hróbjartsson A, Lalu MM, Li T, Loder EW, Mayo-Wilson E, McDonald S, McGuinness LA, Stewart LA, Thomas J, Tricco AC, Welch VA, Whiting P, Moher D. The PRISMA 2020 statement: an updated guideline for reporting systematic reviews. *BMJ* 2021;372:n71. doi:10.1136/bmj.n71
