# Supplementary material for: Transcutaneous auricular vagus nerve stimulation targets the brain-gut axis to treat functional dyspepsia: a scoping review of clinical evidence and mechanistic research
Source: Front Neurosci. 2026 Sep 16;20:1876591. doi: 10.3389/fnins.2026.1876591 (PMC13623837; doi:10.3389/fnins.2026.1876591)
Supplement: Supplementary file 2 [file Table_2.docx]

**Supplementary Material S2**

**Full database search strategies**

*Transcutaneous auricular vagus nerve stimulation targets the brain–gut axis to treat functional dyspepsia: a systematic review of clinical evidence and mechanistic research*

**Date last searched:** 20 December 2025

**Date range:** Inception of each database to 20 December 2025

**Language restriction:** Full text available in English

**Publication-date restriction:** None

**Search strategy structure:** Two concept blocks combined with the Boolean operator AND — (1) intervention concept (transcutaneous auricular vagus nerve stimulation and variants), and (2) condition concept (functional dyspepsia and variants). Within each concept block, controlled vocabulary (MeSH or Emtree) and free-text terms in title/abstract fields were combined with the Boolean operator OR. Truncation symbols (*) and field tags were used as appropriate to each database's syntax.

# **S2.1 PubMed (via NCBI)**

**Interface:** https://pubmed.ncbi.nlm.nih.gov/

**Coverage:** 1946 to 20 December 2025

## **Search lines**

| **#** | **Query** |
| --- | --- |
| **#1** | "transcutaneous auricular vagus nerve stimulation"[Title/Abstract] |
| **#2** | "transcutaneous vagus nerve stimulation"[Title/Abstract] |
| **#3** | "auricular vagus nerve stimulation"[Title/Abstract] |
| **#4** | taVNS[Title/Abstract] |
| **#5** | tVNS[Title/Abstract] |
| **#6** | "vagus nerve stimulation"[MeSH Terms] |
| **#7** | #1 OR #2 OR #3 OR #4 OR #5 OR #6 |
| **#8** | "dyspepsia"[MeSH Terms] |
| **#9** | "functional dyspepsia"[Title/Abstract] |
| **#10** | "non-ulcer dyspepsia"[Title/Abstract] OR "nonulcer dyspepsia"[Title/Abstract] |
| **#11** | "postprandial distress syndrome"[Title/Abstract] |
| **#12** | "epigastric pain syndrome"[Title/Abstract] |
| **#13** | #8 OR #9 OR #10 OR #11 OR #12 |
| **#14** | #7 AND #13 |

**Notes for PubMed:**

• [Title/Abstract] restricts the field search to titles and abstracts. [MeSH Terms] uses the controlled vocabulary.

• PubMed automatically applies term mapping for free-text terms; this is acceptable in our protocol.

• Records identified: see Figure 1 (PRISMA 2020 flow diagram).

# **S2.2 Embase (via Elsevier)**

**Interface:** https://www.embase.com/

**Coverage:** 1947 to 20 December 2025

## **Search lines**

| **#** | **Query** |
| --- | --- |
| **#1** | 'transcutaneous auricular vagus nerve stimulation':ti,ab,kw |
| **#2** | 'transcutaneous vagus nerve stimulation':ti,ab,kw |
| **#3** | 'auricular vagus nerve stimulation':ti,ab,kw |
| **#4** | taVNS:ti,ab,kw |
| **#5** | tVNS:ti,ab,kw |
| **#6** | 'vagus nerve stimulation'/exp |
| **#7** | #1 OR #2 OR #3 OR #4 OR #5 OR #6 |
| **#8** | 'functional dyspepsia'/exp |
| **#9** | 'dyspepsia'/exp |
| **#10** | 'functional dyspepsia':ti,ab,kw |
| **#11** | 'non ulcer dyspepsia':ti,ab,kw OR 'nonulcer dyspepsia':ti,ab,kw |
| **#12** | 'postprandial distress syndrome':ti,ab,kw |
| **#13** | 'epigastric pain syndrome':ti,ab,kw |
| **#14** | #8 OR #9 OR #10 OR #11 OR #12 OR #13 |
| **#15** | #7 AND #14 |

**Notes for Embase:**

• :ti,ab,kw restricts the search to the title, abstract, and keyword fields.

• /exp explodes the Emtree term to capture narrower terms in the hierarchy.

• Single quotation marks delimit multi-word phrases in Emtree/Embase syntax.

# **S2.3 Web of Science Core Collection**

**Interface:** https://www.webofscience.com/

**Indexes searched:** Science Citation Index Expanded (SCI-EXPANDED), Social Sciences Citation Index (SSCI), Emerging Sources Citation Index (ESCI)

**Coverage:** Inception to 20 December 2025

## **Search lines**

| **#** | **Query** |
| --- | --- |
| **#1** | TS=("transcutaneous auricular vagus nerve stimulation") |
| **#2** | TS=("transcutaneous vagus nerve stimulation") |
| **#3** | TS=("auricular vagus nerve stimulation") |
| **#4** | TS=(taVNS) |
| **#5** | TS=(tVNS) |
| **#6** | #1 OR #2 OR #3 OR #4 OR #5 |
| **#7** | TS=("functional dyspepsia") |
| **#8** | TS=("non-ulcer dyspepsia" OR "nonulcer dyspepsia") |
| **#9** | TS=("postprandial distress syndrome") |
| **#10** | TS=("epigastric pain syndrome") |
| **#11** | TS=(dyspepsia) |
| **#12** | #7 OR #8 OR #9 OR #10 OR #11 |
| **#13** | #6 AND #12 |

**Notes for Web of Science:**

• TS= is the Topic field tag (title, abstract, author keywords, Keywords Plus).

• Web of Science does not use a controlled vocabulary equivalent to MeSH; coverage is keyword-based.

# **S2.4 Cochrane Library (CENTRAL)**

**Interface:** https://www.cochranelibrary.com/

**Database:** Cochrane Central Register of Controlled Trials (CENTRAL)

**Coverage:** Inception to 20 December 2025

## **Search lines**

| **#** | **Query** |
| --- | --- |
| **#1** | [mh "vagus nerve stimulation"] |
| **#2** | ("transcutaneous auricular vagus nerve stimulation"):ti,ab,kw |
| **#3** | ("transcutaneous vagus nerve stimulation"):ti,ab,kw |
| **#4** | ("auricular vagus nerve stimulation"):ti,ab,kw |
| **#5** | (taVNS):ti,ab,kw |
| **#6** | (tVNS):ti,ab,kw |
| **#7** | #1 OR #2 OR #3 OR #4 OR #5 OR #6 |
| **#8** | [mh dyspepsia] |
| **#9** | ("functional dyspepsia"):ti,ab,kw |
| **#10** | ("non-ulcer dyspepsia" OR "nonulcer dyspepsia"):ti,ab,kw |
| **#11** | ("postprandial distress syndrome"):ti,ab,kw |
| **#12** | ("epigastric pain syndrome"):ti,ab,kw |
| **#13** | #8 OR #9 OR #10 OR #11 OR #12 |
| **#14** | #7 AND #13 |

**Notes for Cochrane Library:**

• [mh "..."] denotes MeSH heading search in Cochrane Library syntax.

• :ti,ab,kw restricts free-text searches to title, abstract, and keyword fields.

• The Cochrane Central Register of Controlled Trials (CENTRAL) was prioritised for identifying randomized trial reports.

# **S2.5 Additional sources**

***Hand-searching of reference lists.*** The reference lists of all included studies, and of relevant narrative and systematic reviews on taVNS or vagus nerve stimulation in functional gastrointestinal disorders, were screened to identify additional eligible studies.

***Citation searching.*** Forward citation tracking of all included studies was performed in Web of Science.

***Trial registries.*** ClinicalTrials.gov and the Chinese Clinical Trial Registry were consulted to identify completed and ongoing trials whose published reports might have been missed by the database searches.

# **S2.6 Reproducibility statement**

The search strategies above were developed iteratively by the review team in consultation with all reviewers. The strategies are reported in sufficient detail to allow exact reproduction by an independent researcher. Records returned by each search were exported in RIS format to EndNote (Clarivate Analytics, Philadelphia, PA, USA) for deduplication, and to Zotero 5.0 (Corporation for Digital Scholarship, Vienna, VA, USA) for screening. The number of records identified by each database is reported in Figure 1 (the PRISMA 2020 flow diagram).
