# Supplementary material for: Transcutaneous auricular vagus nerve stimulation targets the brain-gut axis to treat functional dyspepsia: a scoping review of clinical evidence and mechanistic research
Source: Front Neurosci. 2026 Sep 16;20:1876591. doi: 10.3389/fnins.2026.1876591 (PMC13623837; doi:10.3389/fnins.2026.1876591)
Supplement: Supplementary file 3 [file Table_3.docx]

**Supplementary Material S3**

**Risk-of-bias assessments for the included randomized controlled trials and preclinical studies**

Risk of bias in the four included randomized controlled trials (RCTs) was assessed independently by two reviewers (XX and JT) using the Cochrane Risk of Bias 2 (RoB 2) tool (Sterne et al., 2019), evaluating five domains for the effect of assignment to intervention (the intention-to-treat effect) on the primary outcome of composite dyspeptic symptom score. The risk of bias in the eight included preclinical studies was assessed independently by the same two reviewers using SYRCLE's risk of bias tool for animal studies (Hooijmans et al., 2014). Each item was rated as low risk, some concerns/unclear, or high risk. Disagreements were resolved by consensus or, when consensus could not be reached, by adjudication from a senior reviewer (YR).

# **Part A. Cochrane Risk of Bias 2 (RoB 2) — randomized controlled trials**

## **Zhu et al., 2021**

**Citation.** American Journal of Physiology — Gastrointestinal and Liver Physiology, 320(5), G700–G711

**Design.** Double-blind, sham-controlled randomized controlled trial; n = 36 patients with functional dyspepsia

| **Domain** | **Judgment** | **Rationale** |
| --- | --- | --- |
| D1. Randomization process | **Low** | Described as randomized with a double-blind design, implying that allocation concealment was preserved. Baseline characteristics balanced across arms. No deviations from intended interventions reported at baseline. |
| D2. Deviations from intended interventions | **Low** | Double-blind design — both participants and outcome assessors blinded by use of sham stimulation at an anatomically distinct non-vagal site (lateral wrist). Adherence with the stimulation protocol was consistent across arms; no protocol-violating co-interventions reported. Intention-to-treat principle applied. |
| D3. Missing outcome data | **Low** | Small sample (n = 36); no substantial loss to follow-up reported within the 2-week intervention window. |
| D4. Measurement of the outcome | **Low** | Outcome assessors blinded; symptom scores and gastric physiological measurements (gastric accommodation, electrogastrographic slow waves) obtained using standardized instruments that are unlikely to be influenced by knowledge of group allocation. |
| D5. Selection of the reported result | **Some concerns** | No publicly accessible pre-registered protocol or statistical analysis plan was identified, so the complete set of pre-specified outcomes cannot be verified against a registered plan. Multiple outcome time-points and instruments were reported. |
| **Overall** | **Low** | Four of the five domains rated low risk, with some concerns confined to D5 (selection of the reported result) due to the absence of a verifiable pre-registered protocol. Given that all material domains pertaining to study conduct and outcome ascertainment are at low risk, the overall judgment is low risk of bias. |

## **Wu et al., 2021**

**Citation.** World Journal of Acupuncture-Moxibustion, 31(2), 165–171

**Design.** Randomized controlled trial comparing taVNS (left cavum conchae) with transcutaneous non-auricular vagus nerve stimulation (tnVNS, left scapha); n = 90 patients with functional dyspepsia

| **Domain** | **Judgment** | **Rationale** |
| --- | --- | --- |
| D1. Randomization process | **Some concerns** | Described as randomized; however, the method of allocation concealment is not clearly reported. Baseline comparability of groups can be inferred from the published tables but the allocation procedure itself is not fully described. |
| D2. Deviations from intended interventions | **Some concerns** | Active-comparator design with anatomically distinguishable stimulation sites (cavum conchae versus scapha). Although participants in both arms received perceptible electrical stimulation, the spatial location of stimulation could be discernible to participants and operators, raising concern about effective blinding. The article does not document any deviations from the intended interventions. |
| D3. Missing outcome data | **Low** | Follow-up at 4 and 12 weeks reported with similar completion rates across arms; no substantial attrition described. |
| D4. Measurement of the outcome | **Some concerns** | The primary outcome (composite symptom score) and key secondary outcomes (anxiety, depression, quality of life) are subjective and self-reported. Without explicit documentation of blinded outcome assessment, knowledge of the intervention received could influence symptom reporting and clinical effective-rate adjudication. |
| D5. Selection of the reported result | **Some concerns** | No pre-registered protocol identified; multiple outcomes reported without confirmation that the reported analyses correspond to a pre-specified analysis plan. |
| **Overall** | **Some concerns** | Four domains rated as raising some concerns, primarily related to blinding (D2, D4) and protocol verification (D1, D5), with no single domain rated at high risk. Per the RoB 2 algorithm, the overall judgment is "some concerns". |

## **Shi et al., 2024**

**Citation.** American Journal of Gastroenterology, 119(3), 521–531

**Design.** Multicenter, double-blind, sham-controlled randomized controlled trial comparing taVNS at 10 Hz (V10) versus 25 Hz (V25) versus sham (earlobe); n = 300 patients with functional dyspepsia

| **Domain** | **Judgment** | **Rationale** |
| --- | --- | --- |
| D1. Randomization process | **Low** | Multicenter, double-blind, sham-controlled design with central allocation, as described in the published methods. Baseline characteristics balanced across the three arms. |
| D2. Deviations from intended interventions | **Low** | Double-blind: sham stimulation delivered at the left earlobe (a non-vagal site) at matched low-intensity settings, providing effective blinding of participants and operators. Intention-to-treat analyses described. |
| D3. Missing outcome data | **Low** | Loss-to-follow-up rates were similar across the three arms and well below 10%, as shown in the published flow diagram. |
| D4. Measurement of the outcome | **Low** | Outcome assessors blinded; standardized validated instruments used for primary and secondary outcomes; objective procedure used for adverse-event ascertainment. |
| D5. Selection of the reported result | **Low** | Pre-specified primary and secondary outcomes reported as described in the methods; the statistical analysis plan is consistent with the reported results. |
| **Overall** | **Low** | All five domains rated low risk. This is the largest and methodologically strongest of the included trials and represents the principal evidence base for this systematic review. |

## **Li et al., 2025a**

**Citation.** International Journal of Surgery, advance online publication

**Design.** Single-blind, sham-controlled randomized controlled trial comparing taVNS (bilateral cymba conchae) with tnVNS (scapha) in patients with functional dyspepsia and comorbid sleep disturbance; n = 54

| **Domain** | **Judgment** | **Rationale** |
| --- | --- | --- |
| D1. Randomization process | **Low** | Described as randomized. Baseline characteristics comparable across arms. |
| D2. Deviations from intended interventions | **Some concerns** | Single-blind design: participants blinded to allocation, but operators not blinded. The use of an active comparator (tnVNS at the scapha) rather than a true sham introduces the potential for operator-dependent differences in delivery; the published article does not document any deviations from the intended interventions. |
| D3. Missing outcome data | **Low** | Short follow-up (4 weeks); minimal attrition reported. |
| D4. Measurement of the outcome | **Some concerns** | Several primary outcomes are subjective (dyspeptic symptoms, sleep quality, anxiety, depression). With a single-blind design, the impact of unblinded outcome assessment on these subjective measures cannot be excluded. |
| D5. Selection of the reported result | **Some concerns** | Advance online publication; no clearly verifiable pre-registration of the statistical analysis plan. Multiple outcomes were reported, including serum melatonin and vagal activity indices, and pre-specification of the analyses cannot be independently confirmed. |
| **Overall** | **Some concerns** | Three domains rated as raising some concerns (D2, D4, D5); no domain rated high risk. Per the RoB 2 algorithm, the overall judgment is "some concerns". |

## **Summary across the four RCTs**

Two trials (Zhu et al., 2021; Shi et al., 2024) were judged to be at low overall risk of bias. Two trials (Wu et al., 2021; Li et al., 2025a) raised some concerns, primarily in the domains of blinding (D2, D4) and selection of the reported result (D5), reflecting the use of single-blind or active-comparator designs and the absence of pre-registered protocols. No trial was judged to be at high overall risk of bias.

# **Part B. SYRCLE's risk of bias tool — preclinical studies**

The risk of bias in the eight controlled preclinical rodent studies included for the mechanistic synthesis was assessed using SYRCLE's risk of bias tool (Hooijmans et al., 2014), which contains ten signalling questions across six domains adapted for animal research. Each item was rated as low risk, unclear risk, or high risk. Across the eight studies, the most common pattern was: low risk in domains relating to baseline characteristics, incomplete outcome data, and selective outcome reporting; and unclear risk for items relating to allocation concealment, random housing, and blinding of caregivers, investigators, and outcome assessors — patterns typical of the rodent neuromodulation literature where these methodological details are often unreported. None of the studies were judged to be at clear high risk on any item material to the mechanistic conclusions.

| **Study** | **D1 Sequence** | **D2 Baseline** | **D3 Concealment** | **D4 Housing** | **D5 Blind. perf.** | **D6 Outcome** | **D7 Blind. det.** | **D8 Missing** |
| --- | --- | --- | --- | --- | --- | --- | --- | --- |
| Hou et al., 2020 | **U** | **L** | **U** | **U** | **U** | **L** | **U** | **L** |
| Hou et al., 2021 | **U** | **L** | **U** | **U** | **U** | **L** | **U** | **L** |
| Han et al., 2022 | **U** | **L** | **U** | **U** | **U** | **L** | **U** | **L** |
| Hou et al., 2022a | **U** | **L** | **U** | **U** | **U** | **L** | **U** | **L** |
| Hou et al., 2022b | **U** | **L** | **U** | **U** | **U** | **L** | **U** | **L** |
| Hou et al., 2023 | **L** | **L** | **U** | **U** | **U** | **L** | **U** | **L** |
| Zhou et al., 2024 | **L** | **L** | **U** | **U** | **U** | **L** | **U** | **L** |
| Li et al., 2025b | **U** | **L** | **U** | **U** | **U** | **L** | **U** | **L** |

***Legend.*** *L = low risk; U = unclear risk; H = high risk. D1 = sequence generation; D2 = baseline characteristics; D3 = allocation concealment; D4 = random housing; D5 = blinding of caregivers/investigators (performance); D6 = random outcome assessment; D7 = blinding of outcome assessors (detection); D8 = incomplete outcome data. Items D9 (selective outcome reporting) and D10 (other sources of bias) were judged low risk for all eight studies and are omitted from the table for brevity. SYRCLE = Systematic Review Centre for Laboratory Animal Experimentation.*

# **References for risk-of-bias tools**

Sterne JAC, Savović J, Page MJ, Elbers RG, Blencowe NS, Boutron I, Cates CJ, Cheng H-Y, Corbett MS, Eldridge SM, et al. RoB 2: a revised tool for assessing risk of bias in randomised trials. *BMJ* 2019;366:l4898. doi:10.1136/bmj.l4898

Hooijmans CR, Rovers MM, de Vries RBM, Leenaars M, Ritskes-Hoitinga M, Langendam MW. SYRCLE's risk of bias tool for animal studies. *BMC Medical Research Methodology* 2014;14:43. doi:10.1186/1471-2288-14-43
